# Research progress of ferroptosis in Alzheimer disease: A review

**DOI:** 10.1097/MD.0000000000035142

**Published:** 2023-09-08

**Authors:** Qi Han, Li Sun, Ke Xiang

**Affiliations:** a Doctor from Changchun University of Chinese Medicine, Changchun City, Jilin Province, China; b Chief Physician of Jilin Academy of Chinese Medicine, Chaoyang District, Changchun City, Jilin Province, China.

**Keywords:** Alzheimer disease, ferroptosis, iron metabolism, lipid peroxidation

## Abstract

Ferroptosis is an emerging form of programmed cell death triggered by iron-dependent lipid peroxidation and reactive oxygen species (ROS). Alzheimer disease (AD), a neurodegenerative disorder, is characterized by the degeneration of nerve cells. Recent research has indicated a significant association between ferroptosis and AD; however, the precise underlying mechanism remains elusive. It is postulated that ferroptosis may impact the accumulation of iron ions within the body by influencing iron metabolism, amino acid metabolism, and lipid metabolism, ultimately leading to the induction of ferroptosis in nerve cells. This article centers on the attributes and regulatory mechanism of ferroptosis, the correlation between ferroptosis and AD, and the recent advancements in the therapeutic approach of targeting ferroptosis for the treatment of AD. These results suggest that ferroptosis could potentially serve as a pivotal focus in future research on AD.

## 1. Introduction

Alzheimer disease (AD) is a neurodegenerative disorder predominantly observed in individuals aged 65 years and above, characterized by the progressive deterioration of memory, language, and cognitive functions.^[1]^ With the deepening of global aging, AD has emerged as the fifth most prevalent cause of mortality globally.^[2]^ The clinicopathological characteristics of AD include the abnormal deposition of β-amyloid protein, neuroinflammation, and hyperphosphorylation of Tau protein, resulting in the formation of neuronal fibrillary tangles.^[3]^ However, the precise etiology and progression of AD have yet to be definitively established, and the majority of clinical trials targeting β-amyloid and Tau proteins have proven unsuccessful. Consequently, further investigation is required to fully elucidate the underlying mechanisms of AD.

Ferroptosis, a form of cell death characterized by the accumulation of intracellular iron as proposed by Dixon,^[4]^ may offer valuable insights in this regard. Ferroptosis, distinct from cell necrosis, apoptosis, and autophagy, is primarily distinguished by the decrease in cell volume and the elevation of mitochondrial membrane density, lacking the typical features of necrosis and apoptosis.^[5]^ Previous investigations^[6]^ have demonstrated the pivotal involvement of ferroptosis in neuronal demise and associated neurological disorders, including Parkinson disease, Huntington disease, and stroke, among others. Recent studies^[7]^ have identified certain attributes of ferroptosis, such as iron dysregulation and reactive oxygen species (ROS) accumulation. Simultaneously, several studies^[8]^ have indicated significant enrichment of differentially expressed genes associated with ferroptosis within the gene set linked to AD following high-throughput sequencing. Numerous studies have implicated ferroptosis as the primary etiological factor underlying AD, closely intertwined with the manifestation of neuronal loss and cognitive decline. This article provides a comprehensive review of the interplay between ferroptosis and the onset as well as pathological characteristics of AD, along with an overview of the advancements made in related research.

## 2. Ferroptosis

Ferroptosis is a distinct form of cell death that differs significantly from other known forms of cell death, including autophagy, apoptosis, and necrosis.^[9]^ In cells undergoing ferroptosis, there is a notable increase in ROS and lipids within the cytoplasm, accompanied by a reduction in mitochondrial volume and an increase in cell membrane density.^[10]^ This iron-dependent and oxidative mode of cell death is initiated when the synthesis of glutathione (GSH) or the activity of the GSH-dependent antioxidant enzyme, GSH peroxidase 4, is inhibited both in vitro and in vivo.^[11]^ However, the metabolic regulatory mechanism of ferroptosis is considerably intricate, and there remain certain mechanisms that have yet to be elucidated. This article will examine the facets of metabolism and lipid peroxidation subsequently.

### 2.1. Disorders of iron metabolism

**Figure 1.**
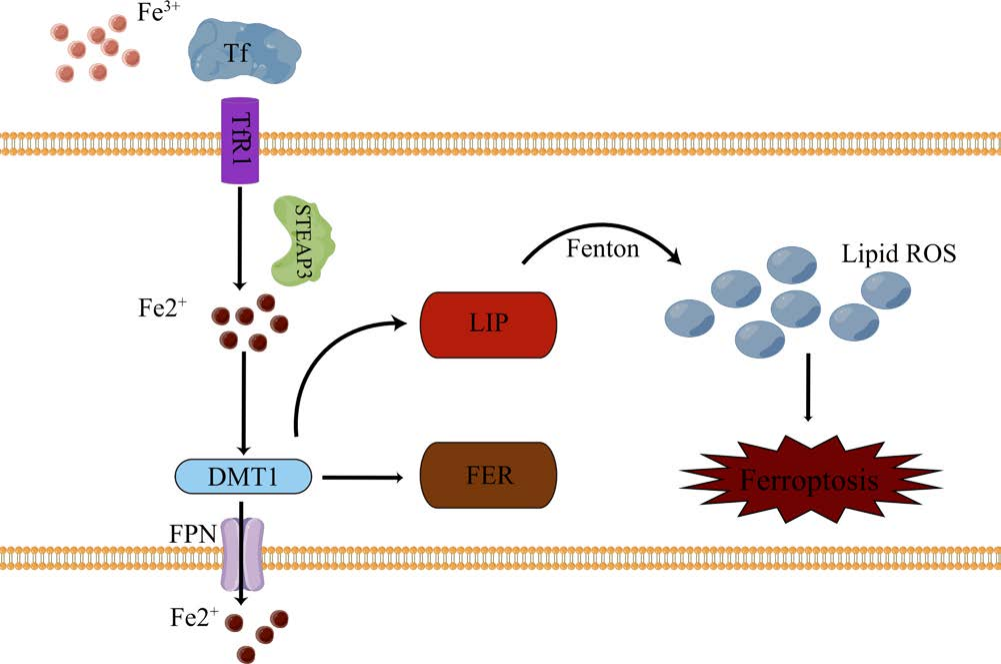
Disorders of iron metabolism induce ferroptosis. Notes: Iron metabolism disorder will lead to an abnormal increase in the LIP. A large number of lipid reactive oxygen species are produced by the Fenton reaction, which destroys cell membranes and induces iron death. DMT1 = divalent metal transporter 1, FER = ferritin, FPN = ferroportin 1, LIP = labile iron pool, ROS = reactive oxygen species, STEAP3 = six-transmembrane epithelial antigen of the prostate 3, Tf = transferrin, TfR1 = transferrin receptor 1.

In normal physiological circumstances, Fe^3+^ in mammalian blood forms a complex with transferrin, which is conveyed into cells via transferrin receptor 1 on the cellular membrane.^[12]^ It is subsequently reduced to Fe^2+^ by divalent metal reductase and either stored in ferritin through metal transporter 1 or expelled by the iron transporter on the cell membrane to uphold iron homeostasis.^[13]^ The disruption of iron metabolism results in an aberrant elevation of labile iron within cells, which subsequently triggers the Fenton reaction, generating a substantial quantity of ROS. This oxidative stress damages the cell membrane and initiates ferroptosis. Consequently, the disorder of iron metabolism is widely acknowledged as the primary mechanism underlying ferroptosis. Additionally, the regulation of ferroptosis involves the IREB2 gene, and the suppression of this gene has been shown to diminish the incidence of ferroptosis. Heat shock protein β1 can reduce the intracellular iron ion concentration by inhibiting the expression of TfR1, so overexpression of heat shock protein β1 can inhibit ferroptosis (Fig. 1).^[14]^

### 2.2. Lipid peroxidation

**Figure 2.**
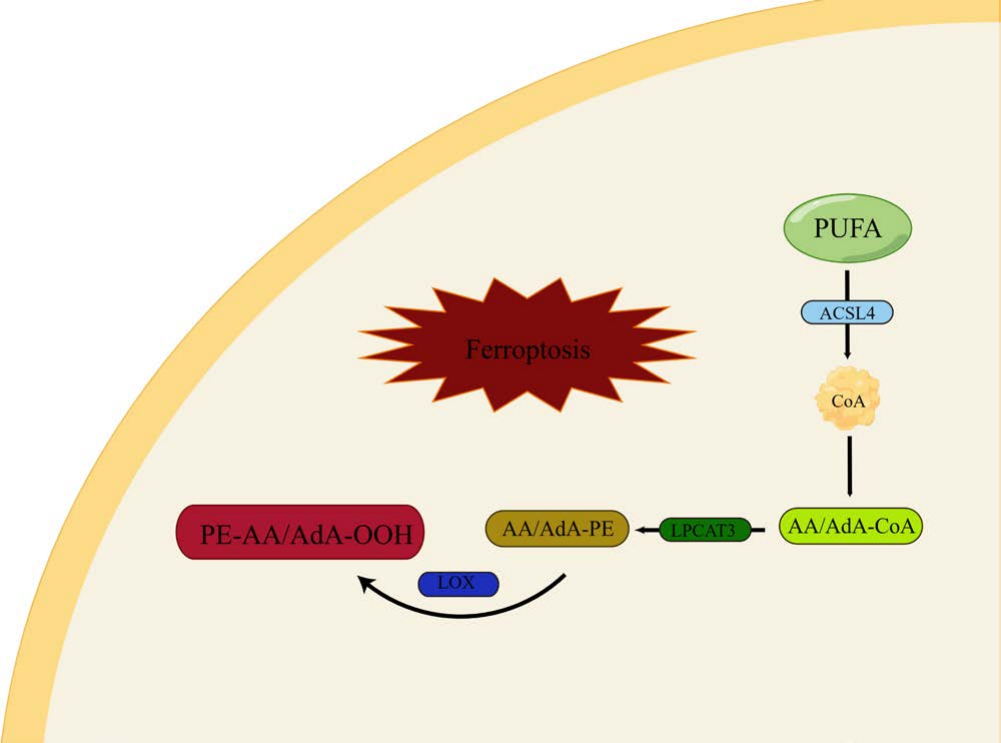
Lipid peroxidation induces ferroptosis. Notes: Lipid peroxidation causes cell damage and activates ferroptosis. ACSL4 = Acyl-CoA synthetase long chain family member 4, CoA = coenzyme A, LOX = lipoxygenase, LPCAT3 = lysophosphatidylcholine acyltransferase 3, PUFA = polyunsaturated fatty acid.

According to relevant studies, it has been demonstrated that polyunsaturated fatty acids (PUFAs) can undergo lipid peroxidation and induce ferroptosis through the action of lipoxygenase (LOX).^[15]^ Specifically, arachidonic acid in PUFA can be catalyzed by acyl-CoA synthetase long-chain family member 4 and conjugated with CoA (coenzyme A) to form the derivative AA/AdA-CoA. Subsequently, this derivative is esterified by lysophosphatidyltransferase 3, resulting in the formation of a peroxide substrate. Oxidation of this substrate leads to the generation of lipid hydroperoxides, causing cellular damage and triggering the activation of ferroptosis (Fig. 2).^[16]^ Acyl-CoA synthetase long-chain family member 4 is widely recognized as the principal enzyme associated with ferroptosis.^[17]^ Additionally, α-tocopherol, an endogenous inhibitor of lipid peroxidation, has been found to impede ferroptosis by intervening in this pathway.^[18]^

Furthermore, numerous studies have elucidated the connection between ferroptosis and ROS. The buildup of ROS within cells is a direct catalyst for ferroptosis, which can be attributed to the Fenton reaction, nicotinamide adenine dinucleotide phosphate-dependent lipid peroxidation, and depletion of GSH. Lysophosphatidylcholine acyltransferase 3 and acyl-CoA synthetase long-chain family member 4 are genes involved in lipid metabolism, and the induction of ferroptosis is attributed to “class 2” FINs.^[19]^ Although the precise mechanism by which iron ions accelerate ferroptosis remains incompletely understood, it has been observed that iron chelators inhibit the donation of electrons from iron ions to oxygen during the process of ferroptosis, resulting in the formation of ROS. Among the various chelators, lipophilic chelators and deferoxamine have garnered significant attention due to their distinct targets in the context of ferroptosis. The LOX family, acting as the co-channel of iron-dependent L-ROS, possesses the ability to impede the degradation of GSH or glutathione peroxidase 4 (GPX4) and hinder ferroptosis.^[20]^ Oxidized PUFAs play a crucial role in LOX, facilitating the conversion of PUFAs into hydroperoxide intermediates, such as HPETE oxidation form AA.^[20]^ LOXs involved in lipid peroxidation necessitate the presence of iron at their active sites for catalytic activity, and the iron chelators deferoxamine and deferiprone effectively inhibit ferroptosis by extracting essential iron from LOXs.^[21]^ arachidonate15-lipoxygenase (ALOX15) plays an important role in elastin-induced ferroptosis by promoting the formation of Lipid-OOH.^[22]^

### 2.3. Amino acid metabolism

GSH, a vital antioxidant within the human body, is involved in various processes facilitated by the cysteine-glutamate transporter. This transporter plays a crucial role in the antioxidant mechanism by converting cystine into cysteine, which serves as the rate-limiting substrate for the synthesis of GSH. Additionally, GSH peroxidase 4 aids in the generation of GSH disulfide through the oxidation of GSH. This process effectively reduces phospholipid hydroperoxides to harmless phospholipid alcohols, thereby mitigating ROS levels and safeguarding cells against potential damage. If the depletion of GSH occurs as a result of cysteine level inhibition, it will lead to a reduction in the antioxidant function of GPX4, resulting in the accumulation of ROS within cells and the initiation of ferroptosis.^[23]^ Conversely, the maintenance of normal GSH levels and GPX4 function in cells can inhibit the occurrence of ferroptosis.^[24]^ Additionally, several studies^[25]^ have demonstrated that the knockout of GPX4 in mice or the direct use of GPX4 inhibitors can enhance lipid peroxidation and intracellular ROS accumulation, suggesting that GPX4 may serve as a crucial regulator of ferroptosis. Therefore, GSH depletion is an important mechanism of ferroptosis, and the decreased GPX4 activity is a representative marker of ferroptosis.

## 3. AD and ferroptosis

Iron is an intrinsic metal ion that plays a crucial role in various essential physiological processes within the brain. Notably, alterations in brain iron levels and distribution in individuals with AD are accompanied by the influence of several pivotal molecules involved in iron transportation, storage, and regulation of iron balance, such as TfR1, Tf, ferritin, and FPN1. These molecules collectively contribute to the onset and progression of AD. The presence and distribution of iron in amyloid-beta (Aβ) plaques and neuronal tangles in the brains of individuals with AD have been extensively documented. As early as 1992, Connor study revealed a significant accumulation of iron within the senile plaques formed by Aβ and the surrounding aggregated cells in brain slices of AD patients, indicating the presence of brain iron deposition and disruption of iron homeostasis in AD.^[26]^ Furthermore, the early stages of AD are characterized by the concurrent accumulation of Aβ and increased iron concentration, suggesting a potentially significant role of iron in the pathogenesis of AD.

### 3.1. Dysregulation of iron homeostasis in AD

The pathogenesis of AD involves a disruption in iron homeostasis, as evidenced by an imaging study that has identified the inferior temporal cortex as the primary brain region affected by iron accumulation in AD.^[27]^ In AD patients, ferritin dysfunction and the formation of protein aggregates occur in the brain. Abnormal ferritin function leads to the release of iron into an unstable ferrous iron pool, which increases susceptibility to ferroptosis.^[28]^ Additionally, research has indicated a correlation between iron homeostasis disorder and the presence of Aβ and tau lesions in AD. The transportation of amyloid precursor protein to the neuronal surface facilitates the stability of iron export protein and transporter protein, resulting in the loss of soluble Tau protein. The iron changes in AD occur as a consequence of protein accumulation and altered iron trafficking related to tau and Aβ.^[29]^ Simultaneously, the cellular iron exclusion is diminished, leading to an augmentation of the redox-active ferrous iron pool. This pool generates hydroxyl radicals through the Fenton reaction, which in turn induces ferroptosis and enhances the neuroinflammatory response by generating ROS.^[8]^ The resulting production of Aβ, down-regulation of FPN1, and iron accumulation gradually form A complex cycle of aggravation in AD patients.

### 3.2. Ferroptosis lipid peroxidation in AD

Lipids constitute a significant constituent of the brain, comprising 40% to 75% of its dry weight and up to 80% of the myelin sheath, which serves a crucial role in energy metabolism and signal transduction.^[30]^ Given the brain physiological functional needs, it possesses a substantial abundance of unsaturated lipids and exhibits a heightened requirement for redox-active metals, rendering it more susceptible to ferroptosis. The elevated levels of free radicals in the brain of individuals with AD create a conducive environment for the onset of lipid peroxidation.^[31]^ In the context of AD, prevailing viewpoints suggest that the generation of pathological Aβ primarily occurs through the ongoing hydrolysis of β-amyloid precursor protein on neuronal cell membranes or peripheral organs within the brain, facilitated by β-secretase and γ-secretase enzymes. Additionally, Aβ that is not synthesized within the brain can infiltrate brain tissue via the bloodstream.^[32]^ Within the brain, Aβ manifests in 3 distinct forms: monomers, oligomers, and filaments, with Aβ oligomers exerting the most pronounced impact on AD pathology.^[33]^ The findings from previous research^[34]^ have demonstrated a significant increase in the levels of lipid peroxides, specifically 4-HNE, in brain regions abundant in Aβ oligomers. This suggests that the presence of Aβ oligomers may have an impact on the extent of lipid peroxidation within the brain. Additionally, other studies^[35]^ have indicated that Aβ oligomers can integrate into lipid bilayers, thereby influencing the efficiency of membrane phospholipid dehydrogenation during the synthesis of free radicals. This, in turn, regulates the initiation of the non-enzymatic process of ferroptosis lipid peroxidation.

There are also some links between lipid peroxidation and Tau protein. The formation of neurofibrillary tangles resulting from the hyperphosphorylation of the Tau protein has long been a focal point of research, and the abnormal phosphorylation of the Tau protein is closely associated with ferroptosis and lipid peroxidation. Several studies^[36]^ have indicated that PUFAs can facilitate the conformational alteration and polymerization of Tau protein, thereby confirming the involvement of lipids in the brain lesions associated with Tau protein in AD patients. Simultaneously, a recent study analyzed cellular and synaptosome lipid rafts extracted from the brains of Aβ amyloid model mice (Tg2576 mice) and double transgenic mice (Tg2576 × TgTauP301L mice). The findings of this longitudinal investigation revealed that lipid rafts serve as a shared platform for the pathological progression of AD.^[37]^ Furthermore, the up-regulation of AMPK, a crucial regulator of ferroptosis lipid peroxidation, not only inhibits phospholipid synthesis but also exerts an inhibitory effect on the phosphorylation of tau protein.^[38]^ Therefore, in addition to being an important marker of ferroptosis, lipid peroxidation also plays a role in AD. Ferroptosis, as an important key to both of them, should be paid more attention to.

### 3.3. AD and GSH depletion

The previous section has provided a comprehensive account of GSH depletion. Inhibition or removal of GSH depletion or GPX4 within the central nervous system has been observed to facilitate the occurrence of ferroptosis, resulting in nerve damage, cognitive dysfunction, neurodegenerative diseases, and potentially AD.^[39]^ Notably, reduced levels of GSH in the hippocampus and frontal cortex have been linked to significant cognitive impairment, indicating the potential of GSH as a biomarker for AD.^[28]^ Previous research^[40]^ has demonstrated that the occurrence of ferroptosis can result in dysfunction of the cystine glutamate transporter receptor, leading to an elevation in extracellular glutamate levels and a reduction in GSH synthesis. This, in turn, contributes to the development of AD through glutamate excitotoxicity. Notably, in the frontal cortex of individuals with early mild cognitive impairment and mild AD, evidence of GSH depletion, as well as reductions in GPX4 and superoxide dismutase, can be observed. These findings suggest that GSH depletion plays a role in the early stages of AD.^[41]^ Several studies^[42]^ have demonstrated that mutations in presenilin, a genetic factor associated with familial AD, can impede the expression of GPX4. This inhibition subsequently exacerbates the deposition of Aβ and the hyperphosphorylation of Tau, ultimately leading to considerable neuronal loss and degeneration of the hippocampus. Concurrently, disturbances in iron metabolism or other mechanisms related to ferroptosis emerge, resulting in a substantial increase in ROS production within the brain. This, in turn, diminishes the levels of GSH and GP4, further hastening the progression of AD.^[43]^

## 4. AD treatment studies based on ferroptosis regulation

**Figure 3.**
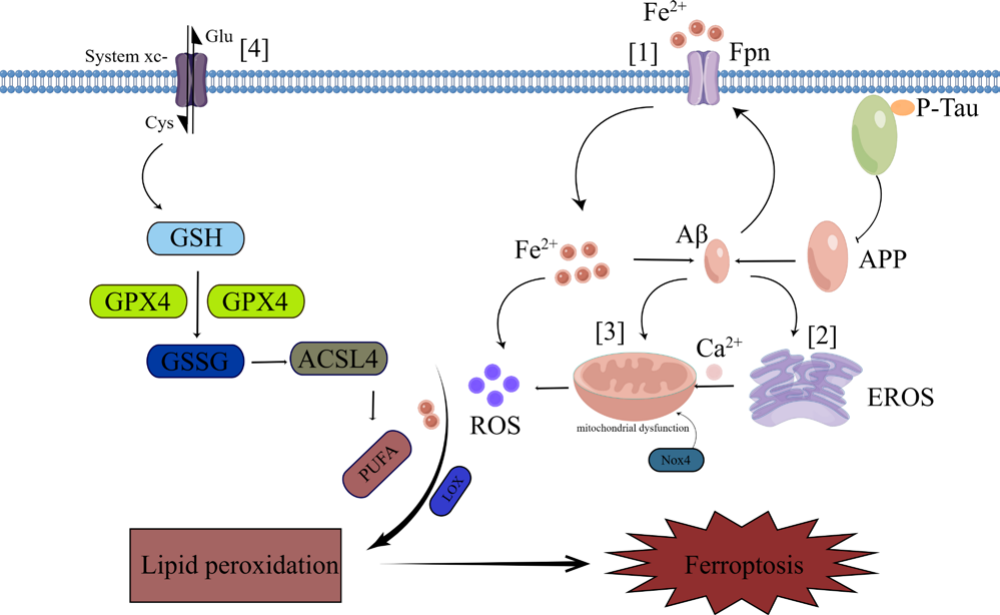
Diagram of the ferroptosis mechanism. Notes: [1] In the brains of individuals with AD, the presence of high concentrations of Aβ can lead to cytotoxicity, resulting in the direct downregulation of FPN1 in primary neurons and the hippocampus. This suggests that a complex vicious cycle is formed, involving Aβ formation, FPN1 downregulation, and iron accumulation in AD. The reduced expression of FPN1 in AD leads to a decrease in cellular iron excretion, causing an increase in the pool of REDOX active ferrous ions. This increase in ferrous ions leads to the generation of hydroxyl radicals through the Fenton reaction, and subsequently, the production of ROS, which further contributes to iron-induced cell death and enhances the neuroinflammatory response. [2] The accumulation of intracellular Aβ has been observed to elicit a stress response in the endoplasmic reticulum, thereby impacting the synthesis of proteins and disulfide bonds, ultimately resulting in protein misfolding. Concurrently, this phenomenon has been found to diminish the cellular levels of the primary endogenous antioxidant, GSH, consequently leading to an elevation in reactive oxygen species within the endoplasmic reticulum. Additionally, it has been observed that this phenomenon can result in a decrease in the cellular concentration of the primary endogenous antioxidant, GSH, thereby inducing a state of stress that promotes the accumulation of ROS within the endoplasmic reticulum. [3] The impairment of mitochondrial function triggers oxidative stress, leading to an excessive generation of ROS. The overexpression of NOX4 further exacerbates this process by diminishing the synthesis of 5 protein complexes involved in the electron transport chain and adenosine triphosphate (ATP) production within astrocytic mitochondria. Consequently, this inhibits mitochondrial respiration and significantly augments the extent of mitochondrial metabolic impairment. The promotion of ROS generation, disruption of mitochondrial function, and inhibition of astrocyte antioxidant mechanisms resulted in the induction of oxidative stress and an escalation in iron-dependent cytotoxicity. [4] System XC− is responsible for the absorption of extracellular cystine and the excretion of intracellular glutamate, which in turn promotes the synthesis of GSH. When the cystine-glutamate reverse transporter is inhibited, it results in a deficiency of cysteine within the cell, reduced synthesis of GSH, and the inactivation of GSH-dependent GPX4. Consequently, this leads to lipid peroxidation and induces iron death. Aβ = amyloid β-protein, EROS = endoplasmic reticulum oxidative stress, GPX4 = glutathione peroxidase 4, GSH = glutathione, GSSG = oxidized glutathione, NOX4 = nicotinamide adenine dinucleotide phosphate oxidase 4, P-Tau = phosphorylated tau protein.

At present, the progress of AD drug development is slow. As mentioned above, iron metabolism, GSH, and lipid metabolism are important pathways to regulate ferroptosis by targeting Aβ or Tau protein, so these pathways may become new targets and new strategies for the treatment of AD (Fig. 3).

### 4.1. Iron chelator regulates ferroptosis to treat AD

Iron chelators can inhibit ferroptosis and thereby restore cellular function, and some iron chelators have been used clinically to alleviate iron accumulation. Related studies^[44]^ have shown that the therapeutic 5-YHEDA factor can cross the blood-brain barrier by connecting the low-density lipoprotein receptor and removing excess iron and free radicals in the brain of aging mice. However, in a clinical trial of AD,^[45]^ although the iron chelator deferiprone significantly slowed down the process of AD, the patients’ cognition or memory did not improve, and they suffered from side effects such as appetite and weight loss.

### 4.2. The intervention of ferroptosis lipid peroxidation in the treatment of AD

Long-term studies on AD have found that the pathogenesis of AD is complex, and it is difficult to achieve satisfactory efficacy with a single target intervention. Therefore, the multi-target intervention of ferroptosis and lipid peroxidation in the treatment of AD is worth exploring. N2L, a novel lipid acid-nicotinic acid dimer, can reduce the production of a variety of lipid peroxides,^[46]^ and thus treat ferroptosis-related AD. Tetrahydroxy stilbene glycoside can activate the GSH/1145·GPX4 signaling axis and enhance the activity of the antioxidant system.^[47]^ Tetrahydroxy stilbene glycoside restores mitochondrial function through several signaling pathways to reverse the damage caused by Aβ. This implies promising candidates for the fight against neurodegenerative diseases, especially AD. CMS121, a derivative of fisetin, can remove peroxidation intermediates such as 4-HNE, and regulate lipid metabolism by inhibiting FASN to prevent several AD-related toxicities and alleviate cognitive decline.^[48]^ Vitamin E and vitamin E metabolites also inhibit LOX and prevent ferroptosis.^[49]^ In 2014, a related clinical trial^[50]^ showed that vitamin E supplementation could delay the progression of the disease in patients with mild to moderate AD.

### 4.3. Treatment of AD by targeting GSH depletion

Compounds SRS16-86 and SRS11-92 have been identified as inhibitors of ferroptosis, and their administration has been found to enhance GPX4 activity, reduce neuronal ferroptosis, and facilitate the repair of damaged synapses and other nerve injuries in AD.^[51]^ Forsythoside A has also been shown to inhibit the occurrence of ferroptosis and neuroinflammation in an AD model of APP/PS1 transgenic mice by increasing the levels of GPX4 and GSH.^[52]^ Additionally, tetrahydroxy stilbene glycoside has been found to inhibit ferroptosis by activating the GSH/GPX4 pathway, thereby alleviating learning and memory impairment in both AD and aged mouse models.^[47]^ Ginkgolide B and salidroside have been shown to decrease oxidative stress and neuroinflammation in models of AD, as well as delay the activation of astrocytes and microglia by up-regulating the expression of GPX4. These findings suggest that Ginkgolide B and salidroside exert neuroprotective effects.^[53]^

Simultaneously, the deprivation of cysteine in system XC− inhibited cells leads to a reduction in GSH levels and the inactivation of GPX4. The supply of cysteine can be obstructed by inhibitors of system XC. The presence of elastin promotes the formation of lipid-OOH through ALOX15, thereby inducing ferroptosis.^[22]^ However, the activation of system XC+ enhances the export of glutamate, activates inhibitory metabotropic glutamate receptors, and subsequently mitigates cognitive impairment caused by excessive glutamate in the thalamocortical cortex. This protective effect may be attributed to the inhibition of ferroptosis.^[54]^

## 5. Summary and Prospect

AD is an intricate and multifactorial condition, and a complete understanding of its mechanism remains elusive. Currently, there is a dearth of efficacious pharmaceutical interventions for its treatment. Ferroptosis, as a novel form of cellular demise, is governed by diverse metabolic pathways. In the forthcoming years, investigating the correlation between ferroptosis and the immune system may offer a fresh vantage point for comprehending the immunological aspects of AD pathogenesis. Additionally, the distinctive gene expression observed in the AD brain may serve as a promising target for further elucidating the mechanisms underlying ferroptosis. Currently, despite the demonstrated efficacy of ferroptosis inhibitors in certain models of AD, there remains a need for further investigation into the intricate association between AD and ferroptosis, as well as the examination of ferroptosis inhibitors throughout the progression of AD. As a novel and promising therapeutic target for AD, ferroptosis holds substantial importance in the prospective clinical management and identification of new drugs for AD. As the comprehension of the mechanism of AD progressively advances, the identification of the pathological network responsible for the simultaneous regulation of multiple targets becomes feasible. This network surpasses the constraints imposed by the influence of a solitary target, thereby offering potential avenues for the radical treatment and prevention of AD.

## Author contributions

**Data curation:** Qi Han.

**Methodology:** Li Sun.

**Project administration:** Li Sun.

**Writing – review & editing:** Qi Han, Ke Xiang.

## References

[1] Scheltens P De Strooper B Kivipelto M. Alzheimer’s disease. Lancet. 2021;397:1577–90. 33667416 10.1016/S0140-6736(20)32205-4 PMC8354300

[2] Pleen J Townley R. Alzheimer’s disease clinical trial update 2019-2021. J Neurol. 2022;269:1038–51. 34609602 10.1007/s00415-021-10790-5

[3] Trejo-Lopez JA Yachnis AT Prokop S. Neuropathology of Alzheimer’s disease. Neurotherapeutics. 2022;19:173–85. 34729690 10.1007/s13311-021-01146-y PMC9130398

[4] Dixon SJ Lemberg KM Lamprecht MR. Ferroptosis: an iron-dependent form of nonapoptotic cell death. Cell. 2012;149:1060–72. 22632970 10.1016/j.cell.2012.03.042 PMC3367386

[5] Mou Y Wang J Wu J. Ferroptosis, a new form of cell death: opportunities and challenges in cancer. J Hematol Oncol. 2019;12:34. 30925886 10.1186/s13045-019-0720-y PMC6441206

[6] Yao MY Liu T Zhang L. Role of ferroptosis in neurological diseases. Neurosci Lett. 2021;747:135614. 33485988 10.1016/j.neulet.2020.135614

[7] Huang L McClatchy DB Maher P. Intracellular amyloid toxicity induces oxytosis/ferroptosis regulated cell death. Cell Death Dis. 2020;11:828. 33024077 10.1038/s41419-020-03020-9 PMC7538552

[8] Bao WD Pang P Zhou XT. Loss of ferroportin induces memory impairment by promoting ferroptosis in Alzheimer’s disease. Cell Death Differ. 2021;28:1548–62. 33398092 10.1038/s41418-020-00685-9 PMC8166828

[9] Hirschhorn T Stockwell BR. The development of the concept of ferroptosis. Free Radic Biol Med. 2019;133:130–43. 30268886 10.1016/j.freeradbiomed.2018.09.043 PMC6368883

[10] Angeli JPF Shah R Pratt DA. Ferroptosis Inhibition: mechanisms and Opportunities. Trends Pharmacol Sci. 2017;38:489–98. 28363764 10.1016/j.tips.2017.02.005

[11] Kim DG Hong YH Shin JY. Pattern of respiratory deterioration in sporadic amyotrophic lateral sclerosis according to onset lesion by using respiratory function tests. Exp Neurobiol. 2015;24:351–7. 26713082 10.5607/en.2015.24.4.351 PMC4688334

[12] Chen K Jiang X Wu M. Ferroptosis, a potential therapeutic target in alzheimer’s disease. Front Cell Dev Biol. 2021;9:704298. 34422824 10.3389/fcell.2021.704298 PMC8374166

[13] Wu J Yang JJ Cao Y. Iron overload contributes to general anaesthesia-induced neurotoxicity and cognitive deficits. J Neuroinflammation. 2020;17:110. 32276637 10.1186/s12974-020-01777-6 PMC7149901

[14] Sun X Ou Z Xie M. HSPB1 as a novel regulator of ferroptotic cancer cell death. Oncogene. 2015;34:5617–25. 25728673 10.1038/onc.2015.32 PMC4640181

[15] Zheng J Conrad M. The metabolic underpinnings of ferroptosis. Cell Metab. 2020;32:920–37. 33217331 10.1016/j.cmet.2020.10.011

[16] Riegman M Sagie L Galed C. Ferroptosis occurs through an osmotic mechanism and propagates independently of cell rupture. Nat Cell Biol. 2020;22:1042–8. 32868903 10.1038/s41556-020-0565-1 PMC7644276

[17] Liang D Minikes AM Jiang X. Ferroptosis at the intersection of lipid metabolism and cellular signaling. Mol Cell. 2022;82:2215–27. 35390277 10.1016/j.molcel.2022.03.022 PMC9233073

[18] Gao M Monian P Jiang X. Metabolism and iron signaling in ferroptotic cell death. Oncotarget. 2015;6:35145–6. 26387139 10.18632/oncotarget.5671 PMC4742090

[19] Xie Y Hou W Song X. Ferroptosis: process and function. Cell Death Differ. 2016;23:369–79. 26794443 10.1038/cdd.2015.158 PMC5072448

[20] Eling N Reuter L Hazin J. Identification of artesunate as a specific activator of ferroptosis in pancreatic cancer cells. Oncoscience. 2015;2:517–32. 26097885 10.18632/oncoscience.160 PMC4468338

[21] Zhou B Liu J Kang R. Ferroptosis is a type of autophagy-dependent cell death. Semin Cancer Biol. 2020;66:89–100. 30880243 10.1016/j.semcancer.2019.03.002

[22] Kagan VE Mao G Qu F. Oxidized arachidonic and adrenic PEs navigate cells to ferroptosis. Nat Chem Biol. 2017;13:81–90. 27842066 10.1038/nchembio.2238 PMC5506843

[23] Lane DJR Metselaar B Greenough M. Ferroptosis and NRF2: an emerging battlefield in the neurodegeneration of Alzheimer’s disease. Essays Biochem. 2021;65:925–40. 34623415 10.1042/EBC20210017

[24] Hayano M Yang WS Corn CK. Loss of cysteinyl-tRNA synthetase (CARS) induces the transsulfuration pathway and inhibits ferroptosis induced by cystine deprivation. Cell Death Differ. 2016;23:270–8. 26184909 10.1038/cdd.2015.93 PMC4716307

[25] Ingold I Berndt C Schmitt S. Selenium utilization by GPX4 is required to prevent hydroperoxide-induced ferroptosis. Cell. 2018;172:409–422.e21. 29290465 10.1016/j.cell.2017.11.048

[26] Connor JR Menzies SL St Martin SM. A histochemical study of iron, transferrin, and ferritin in Alzheimer’s diseased brains. J Neurosci Res. 1992;31:75–83. 1613823 10.1002/jnr.490310111

[27] Antharam V Collingwood JF Bullivant JP. High field magnetic resonance microscopy of the human hippocampus in Alzheimer’s disease: quantitative imaging and correlation with iron. Neuroimage. 2012;59:1249–60. 21867761 10.1016/j.neuroimage.2011.08.019 PMC3690369

[28] Ayton S Wang Y Diouf I. Brain iron is associated with accelerated cognitive decline in people with Alzheimer pathology. Mol Psychiatry. 2020;25:2932–41. 30778133 10.1038/s41380-019-0375-7 PMC6698435

[29] Stockwell BR Jiang X Gu W. Emerging mechanisms and disease relevance of ferroptosis. Trends Cell Biol. 2020;30:478–90. 32413317 10.1016/j.tcb.2020.02.009 PMC7230071

[30] Gonzalez-Riano C Garcia A Barbas C. Metabolomics studies in brain tissue: a review. J Pharm Biomed Anal. 2016;130:141–68. 27451335 10.1016/j.jpba.2016.07.008

[31] Niki E. Lipid oxidation that is, and is not, inhibited by vitamin E: consideration about physiological functions of vitamin E. Free Radic Biol Med. 2021;176:1–15. 34481937 10.1016/j.freeradbiomed.2021.09.001

[32] Wang J Gu BJ Masters CL. A systemic view of Alzheimer disease - insights from amyloid-β metabolism beyond the brain. Nat Rev Neurol. 2017;13:612–23. 28960209 10.1038/nrneurol.2017.111

[33] Selkoe DJ Hardy J. The amyloid hypothesis of Alzheimer’s disease at 25 years. EMBO Mol Med. 2016;8:595–608. 27025652 10.15252/emmm.201606210 PMC4888851

[34] Butterfield DA Boyd-Kimball D. Oxidative stress, amyloid-β peptide, and altered key molecular pathways in the pathogenesis and progression of Alzheimer’s disease. J Alzheimers Dis. 2018;62:1345–67. 29562527 10.3233/JAD-170543 PMC5870019

[35] García-Viñuales S Sciacca MFM Lanza V. The interplay between lipid and Aβ amyloid homeostasis in Alzheimer’s disease: risk factors and therapeutic opportunities. Chem Phys Lipids. 2021;236:105072. 33675779 10.1016/j.chemphyslip.2021.105072

[36] King ME Gamblin TC Kuret J. Differential assembly of human tau isoforms in the presence of arachidonic acid. J Neurochem. 2000;74:1749–57. 10737634 10.1046/j.1471-4159.2000.0741749.x

[37] Kawarabayashi T Nakamura T Sato K. Lipid rafts act as a common platform for amyloid-β oligomer-induced Alzheimer’s disease pathology. J Alzheimers Dis. 2022;87:1189–203. 35431249 10.3233/JAD-215662

[38] Wang L Li N Shi FX. Upregulation of AMPK ameliorates Alzheimer’s disease-like tau pathology and memory impairment. Mol Neurobiol. 2020;57:3349–61. 32519244 10.1007/s12035-020-01955-w

[39] Tan Q Fang Y Gu Q. Mechanisms of modulation of ferroptosis and its role in central nervous system diseases. Front Pharmacol. 2021;12:657033. 34149412 10.3389/fphar.2021.657033 PMC8213017

[40] Zhang L Liu W Liu F. IMCA induces ferroptosis mediated by SLC7A11 through the AMPK/mTOR pathway in colorectal cancer. Oxid Med Cell Longev. 2020;2020:1675613. 32322334 10.1155/2020/1675613 PMC7160732

[41] Ansari MA Scheff SW. Oxidative stress in the progression of Alzheimer disease in the frontal cortex. J Neuropathol Exp Neurol. 2010;69:155–67. 20084018 10.1097/NEN.0b013e3181cb5af4 PMC2826839

[42] Greenough MA Lane DJR Balez R. Selective ferroptosis vulnerability due to familial Alzheimer’s disease presenilin mutations. Cell Death Differ. 2022;29:2123–36. 35449212 10.1038/s41418-022-01003-1 PMC9613996

[43] Yang L Nao J. Ferroptosis: a potential therapeutic target for Alzheimer’s disease. Rev Neurosci. 2022;34:573–98. 36514247 10.1515/revneuro-2022-0121

[44] Zou Z Shao S Zou R. Linking the low-density lipoprotein receptor-binding segment enables the therapeutic 5-YHEDA peptide to cross the blood-brain barrier and scavenge excess iron and radicals in the brain of senescent mice. Alzheimers Dement (N Y). 2019;5:717–31. 31921964 10.1016/j.trci.2019.07.013 PMC6944740

[45] Gleason A Bush AI. Iron and ferroptosis as therapeutic targets in Alzheimer’s disease. Neurotherapeutics. 2021;18:252–64. 33111259 10.1007/s13311-020-00954-y PMC8116360

[46] Peng W Zhu Z Yang Y. N2L, a novel lipoic acid-niacin dimer, attenuates ferroptosis and decreases lipid peroxidation in HT22 cells. Brain Res Bull. 2021;174:250–9. 34171402 10.1016/j.brainresbull.2021.06.014

[47] Gao Y Li J Wu Q. Tetrahydroxy stilbene glycoside ameliorates Alzheimer’s disease in APP/PS1 mice via glutathione peroxidase related ferroptosis. Int Immunopharmacol. 2021;99:108002. 34333354 10.1016/j.intimp.2021.108002

[48] Ates G Goldberg J Currais A. CMS121, a fatty acid synthase inhibitor, protects against excess lipid peroxidation and inflammation and alleviates cognitive loss in a transgenic mouse model of Alzheimer’s disease. Redox Biol. 2020;36:101648. 32863221 10.1016/j.redox.2020.101648 PMC7394765

[49] Hinman A Holst CR Latham JC. Vitamin E hydroquinone is an endogenous regulator of ferroptosis via redox control of 15-lipoxygenase. PLoS One. 2018;13:e0201369. 30110365 10.1371/journal.pone.0201369 PMC6093661

[50] Dysken MW Sano M Asthana S. Effect of vitamin E and memantine on functional decline in Alzheimer disease: the TEAM-AD VA cooperative randomized trial. JAMA. 2014;311:33–44. 24381967 10.1001/jama.2013.282834 PMC4109898

[51] Nebie O Devos D Vingtdeux V. The neuroprotective activity of heat-treated human platelet lysate biomaterials manufactured from outdated pathogen-reduced (amotosalen/UVA) platelet concentrates. J Biomed Sci. 2019;26:89. 31666073 10.1186/s12929-019-0579-9 PMC6822406

[52] Wang C Chen S Guo H. Forsythoside a mitigates Alzheimer’s-like pathology by inhibiting ferroptosis-mediated neuroinflammation via Nrf2/GPX4 axis activation. Int J Biol Sci. 2022;18:2075–90. 35342364 10.7150/ijbs.69714 PMC8935224

[53] Shao L Dong C Geng D. Ginkgolide B protects against cognitive impairment in senescence-accelerated P8 mice by mitigating oxidative stress, inflammation and ferroptosis. Biochem Biophys Res Commun. 2021;572:7–14. 34332327 10.1016/j.bbrc.2021.07.081

[54] Okada M Fukuyama K Kawano Y. Memantine protects thalamocortical hyper-glutamatergic transmission induced by NMDA receptor antagonism via activation of system xc. Pharmacol Res Perspect. 2019;7:e00457. 30784207 10.1002/prp2.457 PMC6323135

